# Perinatal Immunotoxicity: Why Adult Exposure Assessment Fails to Predict Risk

**DOI:** 10.1289/ehp.8566

**Published:** 2005-11-16

**Authors:** Rodney R. Dietert, Michael S. Piepenbrink

**Affiliations:** Department of Microbiology and Immunology, College of Veterinary Medicine, Cornell University, Ithaca, New York, USA

**Keywords:** allergy, atopy, autoimmunity, children’s health, developmental immunotoxicology, immune balance, immunoglobulin E, perinatal risk, safety testing

## Abstract

Recent research has pointed to the developing immune system as a remarkably sensitive toxicologic target for environmental chemicals and drugs. In fact, the perinatal period before and just after birth is replete with dynamic immune changes, many of which do not occur in adults. These include not only the basic maturation and distribution of immune cell types and selection against autoreactive lymphocytes but also changes designed specifically to protect the pregnancy against immune-mediated miscarriage. The newborn is then faced with critical immune maturational adjustments to achieve an immune balance necessary to combat myriad childhood and later-life diseases. All these processes set the fetus and neonate completely apart from the adult regarding immunotoxicologic risk. Yet for decades, safety evaluation has relied almost exclusively upon exposure of the adult immune system to predict perinatal immune risk. Recent workshops and forums have suggested a benefit in employing alternative exposures that include exposure throughout early life stages. However, issues remain concerning when and where such applications might be required. In this review we discuss the reasons why immunotoxic assessment is important for current childhood diseases and why adult exposure assessment cannot predict the effect of xenobiotics on the developing immune system. It also provides examples of developmental immunotoxicants where age-based risk appears to differ. Finally, it stresses the need to replace adult exposure assessment for immune evaluation with protocols that can protect the developing immune system.

The premise of this review is that the developing immune system represents a particularly sensitive xenobiotic target that is not effectively modeled through routine screening for immunotoxicity using adult exposure. Hence, adult exposure testing for immunotoxicity is limited in application, cannot address the most significant immune vulnerabilities, and should be replaced with a more predictive assessment protocol. This conclusion is drawn from recent developmental immunotoxicity findings, including those from our own laboratory, as well as from the conclusions of numerous conferences and workshops. These sources point to the special vulnerabilities of the perinatal immune system compared with the fully matured and dispersed immune system of the adult.

Individuals in early-life stages have been recognized as a special subset of the population that is likely to be at greater toxicologic risk than adults (Daston et al. 2004; Kimmel 2005; Landrigan et al. 2004; Selevan et al. 2000). Within this broader framework, a significant number of recent review and consensus workshop reports have stressed that early-life exposure to xenobiotics poses the greatest environmental risk for the immune system and would be expected to exert the greatest effect on subsequent human health (Dietert 2005; Dietert et al. 2000, 2002; Holladay 1999; Holladay and Smialowicz 2000; Holsapple et al. 2005; Kimmel et al. 2005; Luebke et al. 2006; Luster et al. 2005; Van Loveren and Piersma 2004). Yet, immune-associated safety from problematic exposure to environmental chemicals as well as drugs has hinged on adult exposure assessment [Hinton et al. 2000; Luster et al. 1992 U.S. Food and Drug Administration 1999]. Developmental immunotoxicity screening was not included in one recent immunotoxicity draft guidance covering human pharmaceuticals (U.S. Food and Drug Administration 2004), although its potential application within safety screening is under ongoing consideration (Holsapple et al. 2005; Ladics et al. 2005).

In this review we highlight some novel processes of perinatal immune development that both contribute to the immunotoxic vulnerability of the developing immune system and cannot be effectively examined via current adult-exposure assessment. Additionally, specific examples of the problems associated with reliance on adult-induced immunotoxicity assessment are shown for a variety of immunotoxicants.

## Perinatal Immune Development versus the Adult Immune System

Immune development has been characterized from a toxicologic perspective through a series of discrete functional changes representing critical windows of differential vulnerability to toxicants (Dietert et al. 2000; Holsapple et al. 2003; Landreth 2002; Landreth and Dodson 2005; Leibnitz 2005). These reviews have emphasized that each “window” of development likely has different immunologic risks associated with immunotoxicant exposure, and indeed, examples of differential immunotoxic outcomes among these windows do exist (Bunn et al. 2001b; Lee et al. 2001). Although it is not practical or necessary to directly evaluate the differential risk of limited exposures over different periods within perinatal development, it is important to accurately estimate immunologic risk across the entire period of immune development because of the important role of effective immune function in children’s health (Daston et al. 2004; Kimmel et al. 2005).

Table 1 draws upon the broader critical windows of immune development (Dietert et al. 2000) to illustrate a set of seven discrete events that are either unique to perinatal immune development or critical to the post-natal immune integrity while serving a different role in the adult. These immune events include those that are restricted solely to the immune system as well as some involving the role of the immune system in host organ/tissue homeostasis. In most cases, clear associations exist between exposure to specific toxicants and disruption of the perinatal event. It is not simply by chance that this set of early immune events seems to impinge primarily on the risk of atopy, autoimmune disease, and later-life immune balance (which also influences risk of cancer, etc.). In fact, the perinatal period of immune development is precisely the period in which immune balance must shift from that of an allogeneic yet full-term fetus to that of an offspring ready to meet the complete spectrum of disease challenges. At the heart of the issue is the fact that impact of a xenobiotic on that shift cannot be tested with adult exposure assessment.

## Establishing and Renewing Macrophage-Derived Cells in Critical Tissues

One of the early events connecting the immune system to virtually all organs is the differentiation and seeding of myelomonocytic lineage macrophages and macrophage-derived cells to various sites, including the bronchial (e.g., alveolar macrophages), hepatic (Kupffer cells), neurologic (microglia), and reproductive systems (testicular macrophages). These cells provide regulatory and host defense roles in these tissues. Specific examples describe the vulnerability of these tissues during the perinatal period when exposure to toxicants impairs macrophages, including the possibility that the heavy metal lead can impair both the function and the self-renewal of testicular macrophages, which contributes to male sterility problems (Pace et al. 2005). Similarly, pulmonary and alveolar macrophages play a key role in lung development (Beyea et al. 2005), and sensitivity of the perinatal lung to some environmental agents is directly related to alterations in early-life macrophage populations (Cao et al. 2004; Li et al. 2001). In the brain, inappropriate cytokine production from microglial cells and/or astrocytes is now recognized as an early component of many postnatal neurologic diseases (Bell and Hallenbeck 2002; Cacci et al. 2005; Mesples et al. 2005; Ravizza et al. 2005). With Kupffer cells in the liver (Naito et al. 1997), researchers recently found that their capacity to develop a tolerance for lipopolysacharride (LPS) (Uhrig et al. 2005) is critical for the ability of the liver to control inflammation.

### Lymphoid seeding of the thymus and thymopoiesis.

Another early immune process critical for subsequent host defense is the migration of pro–T lymphocytes to the thymus and their expansion during thymopoiesis. During the perinatal period, the thymus is central to the production of T lymphocytes. Even in children, the thymus continues to play the major role in T-lymphocyte generation (Mackall et al. 1995; Schonland et al. 2003). In contrast, the thymus has a much different role in the adult. Although the adult thymus retains some capacity for the production of cells, particularly with severe immune depletion, its role remains minor in the repopulation of T lymphocytes (Hakim et al. 2005; Petrie 2002). Instead, most T-lymphocyte production comes from the periphery in adults (Hakim et al. 2005). The ramifications of this are that the targeting of pro–T lymphocytes by chemicals or drugs and/or induction of thymus atrophy would be expected to have different consequences depending upon age. As shown in Table 1, several environmental agents appear to target either pro–T lymphocytes or the thymopoiesis process.

### Negative selection in the thymus of auto-reactive T-cell clones.

A third early immune process critical for host integrity is the negative selection and removal of autoreactive T-lymphocyte clones in the thymus. This occurs during the process sometimes designated as “T-cell education.” Meylan et al. (2005) provided a clear demonstration of this process as it occurs in humans. Partially mature thymocytes undergo negative selection at the corticomedullary boundary and in the medulla of thymus (Sprent and Kishimoto 2002). This process is essential if self-reactive clones are to be eliminated before birth. Conversely, excessive loss of thymocytes during negative selection leads to T-cell depletion. Although explicit examples of chemicals or drugs blocking negative selection have yet to be determined, this perinatally unique stage of immune development would appear to be a significant factor in later-life autoimmune disease. Metals represent one category of immunotoxicants warranting examination based on the capacity of mercury and other metals to either trigger or accelerate the progression of autoimmune manifestations (Fournie et al. 2002; Lawrence and McCabe 2002; Rowley and Monestier 2005). Conversely, some chemicals are known to disrupt the negative selection process by overpromoting negative selection and inappropriate thymocyte apoptosis. 2,3,7,8-Tetrachlorodibenzo-*p*-dioxin (TCDD) is an example of such a toxicant (Camacho et al. 2004; Fisher et al. 2004).

## Thymic Generation of Regulatory T-Cells (CD4^+^CD25^+^ High Expression) and Acquisition of Tolerance

A parallel protection against self-reactivity resides in the gestational thymic production and subsequent activation of a specialized population of regulatory T lymphocytes. The specialized regulatory T-cell (Treg) population carries the phenotype FoxP3^+^CD4^+^CD25^+^ and develops during gestation (~ 12–13 weeks) in the human fetus (Cupedo et al. 2005; Darrasse-Jeze et al. 2005). Tregs are critical in the suppression of autoreactive lymphocytes that have escaped elimination through negative selection in the thymus (Cupedo et al. 2005; Kronenberg and Rudensky 2005; Sakaguchi and Sakaguchi 2005). It appears that Tregs acquire their regulatory and suppressive phenotype while within the fetal thymus and are further activated in peripheral lymphoid organs (Cupedo et al. 2005). The process through which these cells emerge and acquire the capacity to identify and suppress self-reactive lymphoid clones occurs embryonically. As with negative selection discussed previously, the active perinatal process of producing and activating Tregs is a logical stage where toxicant-induced risk of later-life autoimmune disease would be great. Evidence suggests that some doses of cyclophosphamide (Lutsiak et al. 2005) and cyclosporin A (Kawai et al. 2005) cause inhibition of Treg populations. Treg suppression is thought to be one route to increase allergic disease (Robinson et al. 2004), and the breaking of tolerance to nickel has been associated with Treg suppression (Cavani 2005).

### Maturation of dendritic cells from the fetal (immature) phenotype.

Dendritic cells are known to be important in antigen presentation and in determining the nature of subsequent immune responses. A fifth perinatal process involves the maturation of immature dendritic cells to produce interleukin (IL-12) (counterbalancing IL-10 production) and acquire the capability of promoting T-helper 1 (T_H_1) or type 1 responses. This does not happen in humans until parturition under normal circumstances (Holt and Jones 2000; Holt and Sly 2002).

In fact, the T_H_1 response must be suppressed until after birth to protect the pregnancy from T_H_1-mediated immunologic rejection (Lim et al. 2000). One of the processes for accomplishing this is the metabolism of the amino acid tryptophan by the enzyme indoleamine-2,3-dioxygenase (IDO) to produce tryptophan metabolites such as kynurenines (Fallarino et al. 2003; Gutierrez et al. 2003; Meisel et al. 2004; Mellor et al. 2002). These metabolites selectively suppress T_H_1 function by inducing apoptosis in T_H_1 but not T_H_2 cells (Fallarino et al. 2003), thereby skewing responses toward T_H_2. In the fetus, this is required to avoid allogeneically induced miscarriage (Mellor et al. 2002). But in the newborn this must be corrected to provide adequate immune balance. Not surprisingly, imbalances in IDO activity have been associated with diseases such as colitis (Gurtner et al. 2003) and inflammatory bowel disease (Wolf et al. 2004).

The perinatal system is exquisitely sensitive to these time/life-stage–dependent shifts in immune balance. For example, Malamitsi-Puchner et al. (2005) demonstrated that even the mode of birth delivery can influence the acquisition of T_H_1 cytokine production capacity in humans. Newborns delivered by cesarean section remained more T_H_2 skewed compared with vaginally delivered newborns. This emphasizes the potential problems in using an adult exposure assessment protocol for immunotoxicity to model the perinatal immune changes surrounding birth.

In keeping with this idea, a recent study demonstrated that human cord blood–derived dendritic cells respond completely differently than their adult counterparts when exposed to dexamethasone (Mainali and Tew 2004). Dexamethasone exposure of these immature cells prevents their maturation to promote T_H_1 responses and locks in the T_H_2 IgE-promoting phenotype. The T_H_2 skewing effect appears to be long-lasting (Mainali et al. 2005). This type of early-life-stage–restricted immunotoxicity appears to contribute to an increased risk of atopy and asthma. Andersson et al. (2004) showed that maturation of newborn immature dendritic cells with LPS reduced the development of a T_H_2-associated birch allergen response. In contrast, the lack of dendritic cell maturation from the fetal immature stage was associated with children at risk for type 1 diabetes (Skarsvik et al. 2004). In addition to dexamethasone (Mainali and Tew 2004), nicotine (Nouri-Shirazi and Guinet 2003) has been reported to block dendritic cell maturation. Again, such toxicant-induced perinatal alterations cannot be examined with adult-only exposure because extensive dendritic cell maturation would have occurred after birth and before adult exposure to the test xenobiotic.

### Shifting T_H_ balance for later life.

Beyond dendritic cells, some xenobiotics such as the heavy metals and tryptophan metabolites may directly affect T_H_ cells and contribute to skewed immune responses in later life. Because mammals are born with a T_H_2-skewed functional capacity (Protonotariou et al. 2003), perinatal versus adult exposure assessment actually measures two different alterations. In the perinatal case, the issue is whether a xenobiotic locks in the already existing T_H_2 bias among T lymphocytes, thereby preventing the genetically influenced adult balance to be achieved postnatally. This would allow as the default an increased risk of neonatal T_H_2-associated diseases (Holt and Sly 2002). In contrast, under the best circumstances adult exposure assessment could measure only whether a xenobiotic selectively impaired T_H_1 cells in an already balanced system. At physiologic levels of exposure, many more environmental factors may be capable of delaying or reducing the efficiency of perinatal T_H_1 maturation (thereby perpetuating the fetal imbalance) than can clinically alter adult T_H_ balance.

### Surfactant modulation of macrophages.

Beyond the gestational seeding of macrophages to different tissues and initial maturation *in situ*, there is a special perinatal maturation of macrophages (particularly alveolar) that enables them to acquire increasing host defense capabilities (phagocytosis, chemotaxis, tumor necrosis factor-α production, antibody-dependent cellular cytotoxicity) with increased postnatal age (Goldman et al. 2004). Hence, perinatal exposure to chemicals and drugs would target functionally immature cells in a manner unlike the fully functional population in exposed adults. Among the critical factors in this perinatal macrophage maturation is exposure to various factors known as “collectins” (surfactant proteins).

Although collectins can be immunomodulatory for adult alveolar macrophages (Hickling et al. 2004), they seem to provide important maturational signals for perinatal macrophages that go well beyond defense of the lung (Mendelson and Condon 2005). Palaniyar et al. (2005) discussed the role of surfactant protein D in enhancing macrophage clearance of DNA and in minimizing anti-DNA antibody production. Additionally, Brinker et al. (2001) demonstrated that surfactant interactions with macrophages and dendritic cells help to shift responses from purely innate to acquired immune responses. Surfactant protein A signals amniotic fluid macrophages to migrate to the uterus and initiate the parturition process (Condon et al. 2004; Mendelson and Condon 2005). Complicating the age issue is the fact that surfactant content varies with age (Egberts et al. 1992). Obviously, such perinatal alterations in macrophage activities are difficult to evaluate using adult-only exposure to potential immunotoxicants.

Although these developmental immune events illustrate the biological problem with modeling immunotoxicologic risk using adult exposures, the resulting underestimation of perinatal sensitivity can take several forms. These are described in the following section.

## Nature of Increased Perinatal Immunotoxic Sensitivity

### Dose sensitivity.

The increased sensitivity of the developing versus the adult immune system to immunotoxic alteration can take several forms. First, early-life stages have increased dose sensitivity to most toxicants. There are several examples suggesting that the developing immune system is altered by significantly lower doses of toxicants than those required to produce effects in the adult. Such comparisons were recently reviewed in Luebke et al. (2006). Lead (Chen et al. 2004; Heo et al. 1996; McCabe et al. 1999; Miller et al. 1998; Snyder et al. 2000) appears to differ across ages for immunotoxic end points ranging from 3- to 12-fold in lowest observed adverse effect levels (LOAELS). Similarly, mercury appears to have age-based differences (Havarinasab et al. 2004; Hultman and Hansson-Georgiadis 1999; Silva et al. 2005). With TCDD the age-based range in LOAELS appears to be even greater (Gehrs and Smialowicz 1997, 1999; Gehrs et al. 1997; Smialowicz et al. 1994; Walker et al. 2004; see also Table 2).

### Range and severity of effects.

A second measure of differential age-based sensitivity to immunotoxicants concerns the spectrum and severity of effects. Not surprisingly, immunotoxicants frequently produce a different and unpredictable array of alterations when the exposure occurs *in utero* or in the early neonate versus the adult. Among those immunotoxicants that produce different ranges or severities of outcomes depending upon age of exposure are lead (Bunn et al. 2001b; Lee et al. 2001), methoxychlor (White et al. 2005), T-2 toxin (Holladay et al. 1993b), benzo[*a*]pyrene (Holladay and Smith 1994; Rodriguez et al. 1999), chlordane (Barnett et al. 1985), 7,12-dimethylbenz[*a*]anthracene (Cooray and Jonsson 1990; Holladay and Smith 1994; Holladay et al. 1995), ethanol (Giberson and Weinberg 1995, 1997; Giberson et al. 1997), nonylphenol (Karrow et al. 2004), tributyltins (Smialowicz et al. 1989; Tryphonas et al. 2004; Vos et al. 1990), and genistein (Guo et al. 2002).

For example, with methoxychlor exposure of rats, F_1_ males had significantly elevated levels of splenic antibody-forming cells, unlike their exposed mothers, whereas F_1_ females had a significantly reduced percentage of CD8^+^ T cells (at all doses examined) with no corresponding effect in the exposed dams (White et al. 2005). Likewise with genistein exposure, exposed F_0_ rat dams displayed altered natural killer (NK) activity, whereas their daughters exposed *in utero* had altered antibody-forming cell activity but no change in NK activity (Guo et al. 2002). With TCDD exposure in rats to assess persistent effects, exposed offspring had a significant reduction in contact hypersensitivity with no effect in the exposed dams (Walker et al. 2004). These examples illustrate that adult exposure assessment is inherently ineffective in predicting the range of likely immunotoxic effects after *in utero* exposure.

### Persistence of effects.

Another feature of developmental immunotoxicity is that alterations after early exposure are frequently persistent and last long after exposure, frequently into adulthood of the exposed offspring. Examples where early xenobiotic exposure results in a greater persistence of effects than would be predicted from adult exposure assessment are found with diethylstilbestrol (DES) (Fenaux et al. 2004; Holladay et al. 1993a; Kalland and Forsberg 1978; Luster et al. 1979, 1980) and cyclosporin A (Hussain et al. 2005b).

### Latency.

Finally, one of the anomalies of early exposure is that a sublethal exposure to a toxicant may produce an unrecognizable immunotoxic alteration until the postnatal immune system is placed under subsequent stress. This hidden or cryptic state is referred to as “latency.” A classic example exists for early exposure to DES (Fenaux et al. 2004). In this case, an apparently innocuous early exposure to DES alters the immune system in such a manner that it responds to a second adult estrogenic exposure (which of itself has no effect) with a completely aberrant cytokine production profile. The *in utero* exposure to DES primes the immune system for postnatal unpredictable responses. A similar example has been seen after low-level exposure to lead where postnatal viral infection resulted in unpredictable alterations in leukocyte mobilization (Lee et al. 2002). Obviously, adult exposure assessment affords no opportunity to examine this phenomenon of embryonic-induced immune latency.

## Sex Differences in Sensitivity Outcome

Differential immunotoxic effects between sexes are neither universal after early exposure (Voderstrasse et al. 2004) nor unique to early-life stages. However, a surprising number of examples exist in which males and females have different immune outcomes after perinatal xenobiotic exposure. Xenobiotics can have different effects on the developing immune system based on the hormonal environment (Hussain et al. 2005a). Among the chemicals listed in Table 2, gender differences in developmental immunotoxicity have been reported for lead (Bunn et al. 2001a, 2001b, 2001c; Miller et al. 1998), mercury (Silva et al. 2005), genistein (Guo et al. 2002), nonylphenol (Karrow et al. 2004), TCDD (Gehrs and Smialowicz 1999), and methoxychlor (White et al. 2005).

## Need for Nonadult Exposure Assessment

Table 2 lists examples of immunotoxicants where age-based comparisons exist and adult exposure assessment is not predictive of perinatal sensitivity to the xenobiotic. Although basic hazard identification could be performed on most of the toxicants listed using only adult exposure data, there would be little guidance for protecting early life stages from problematic exposure of the developing immune system. This is one reason that several recent reviews have suggested the benefit of exposure regimes that include all nonadult (conception, gestation, lactation, juvenile) stages of development (Holsapple et al. 2003; Kimmel et al. 2005; Luster et al. 2005). Recent findings of key maturation events surrounding birth and of chemical- and drug-induced disruption of those immune-associated events (Mainali and Tew 2004; Mainali et al. 2005; Shaheen et al. 2005) are further indications that adult-only exposure protocols are unlikely to accurately predict the risk of perinatal immunotoxicity. Exposure over the nonadult stages of immune development is more likely to include those age-based populations at greatest risk.

## Conclusions

Many critical processes occurring during peri-natal immune development are either nonexistent or comparatively unimportant in the adult (e.g., Table 1). Therefore, when safety limits are established on the basis of adult immune exposure data, they likely have limited use for predicting developmental immunotoxicity and protection of the nonadult. For the chemicals and drugs compared across age groups to date, the developing immune system has a greater sensitivity than that of the fully matured adult. Because this increased sensitivity can take different forms (e.g., Table 2), use of magnitude safety factors is of limited benefit in the absence of relevant exposure data. Where adult exposure protocols are used as the only yardstick of immunotoxic safety, consideration should be given to replacing these protocols with exposure regimes extending throughout the nonadult period of development. Given the specific pattern of perinatal immune alterations, it is likely that they explain, in part, the increased incidence of such human diseases as atopy, asthma, and certain autoimmune manifestations.

## Figures and Tables

**Table 1:** Immune toxicant targets associated with perinatal development.

Key perinatal immune events	Timing in humans	Benefit to host	Examples of concern	Health ramifications	Key references
Differentiation and seeding of macrophages to tissues	6–24 WG	Self-renewing populations of microglia, Kupffer cells, and alveolar macrophages; resident macrophage functioning in tissues (e.g., testis)	Lead, LPS, ozone, cyclophosphamide	Inflammation of lung, brain, or liver tissue dysfunction (e.g., male infertility)	Cao et al. 2004; Hao et al. 2001; Janossy et al. 1986; Pace et al. 2005
Seeding of thymus by pro–T cells and thymopoiesis to expand populations	Seeding 8–12 WG, massive expansion of populations 14–26 WG	Production of T-cell clones necessary to establish peripheral T-lymphocyte populations	PAHs, T-2 toxin, tributyltins, TCDD	Thymic atrophy, decreased postnatal T cells and T-dependent function, increased risk of cancer and infectious diseases	Gehrs and Smialowicz 1997; Holladay and Smith 1994, 1995; Holladay et al. 1993b, 1995; Smialowicz et al. 1989, 1994; Vos et al. 1990; Walker et al. 2004
Negative selection and apoptosis of autoreactive thymocytes	15–26 WG	Elimination of most peripheral T-lymphocyte clones	TCDD promotes unnecessary negative thymocyte selection increasing apoptotic cell death	If promoted, then deceased numbers of thymocytes. If impaired, then, increased risk of later-life self- reactivity	Camacho et al. 2004; Fisher et al. 2004
Treg cell (CD4^+^CD25^+^ high) population generation in thymus, seeding and activation in periphery	Thymus appearance 12–13 WG; periphery 14–16 WG	Active suppression of postnatal autoreactive T-cell clones	Possible low-dose cyclophosphamide, selected doses of cyclosporin A	If excessively promoted, then possible immune suppression. If impaired, then increased risk of later autoimmunity or allergy (e.g. breaking tolerance to nickel)	Cavani 2005; Cupedo et al. 2005; Darrasse-Jeze et al. 2005; Kawai 2005; Lutsiak et al. 2005; Robinson et al. 2004; Valmori et al. 2005
Perinatal dendritic cell maturation to support T_H_1 responses	Birth–juvenile	Increase in dendritic cell maturation and T_H_1-promoting capacity after birth to achieve necessary T_H_1 balance	Dexamethasone, nicotine	Increased risk of allergy and some forms of autoimmunity (e.g., type 1 diabetes)	Andersson et al. 2004; De Wit et al. 2003; Krumbiegel et al. 2005, Mainali and Tew 2004; Nouri-Shirazi and Guinet 2003; Renkl et al. 2005; Skarsvik et al. 2004
Increase in T_H_1 response capacity among peripheral T lymphocytes after birth	Birth–juvenile	Needed to avoid life-long T_H_2 skewing	Lead, mercury, kynurenines selectively impair T_H_1 cells, 1-methyl-tryptophan may promote T_H_1	With depressed T_H_1, increased risk of T_H_2 associated diseases such as atopy and asthma	Bunn et al. 2001b, 2001c; Fallarino et al. 2003; Mellor et al. 2002; Miller et al. 1998; Silva et al. 2005; Snyder et al. 2000
Maturation and regulation of fetal macrophages via interactions with surfactants A and D and glutathione sources	16 WG neonatal period SP-D; 19 WG neonatal period SP-A	Needed to avoid oxidative damage to lung and increased risk of respiratory disease; needed to facilitate parturition, needed to regulate macrophages	Ethanol	Increased risk of childhood respiratory disease; potential problems with labor, increased risk of autoimmune disease	Gauthier et al. 2005; Kaneko et al. 2001; Palaniyar et al. 2005; Pryhuber et al. 1991; Seppanen et al. 2005; Zimmermann et al. 2005

Abbreviations: LPS, lipopolysaccharide; PAHs, polycyclic aromatic hydrocarbons; SP-A, surfactant protein A; SP-D, surfactant protein D; T, thymic derived; TCDD, 2,3,7,8-tetra-chlorodibenzo-*p*-dioxin; T_H_1, T helper 1; T_H_2, T helper 2; Treg, T regulatory; WG, weeks of gestation.

**Table 2:** Examples of perinatal-induced immune outcomes not predicted by standard adult-exposure assessment.

Chemical/drug	Nature of age-based difference	Reference(s)
Benzo[*a*]pyrene	Severity of effects (e.g., impact of fetalthymic atrophy)	Holladay and Smith 1994; Lummus and Henningsen 1995; Rodriguez et al. 1999; Wolisi et al. 2001
Chlordane	Dose sensitivity, spectrum of effects	Barnett et al. 1985; Blaylock et al. 1990; Spyker-Cranmer et al. 1982; Theus et al. 1992
Cyclosporin A	Persistence of effects	Hussain et al. 2005b
Dexamethasone	Dose sensitivity Spectrum of effects	Dietert et al. 2003; Mainali and Tew 2004
Diazepam	Dose sensitivity Spectrum/severity of effects	Descotes et al. 1982; Schlumpf et al. 1989
DES	Persistence of effects Latency	Fenaux et al. 2004; Kalland and Forsberg 1978; Luster et al. 1980
7,12-Dimethybenz[*a*]anthracene	Severity of effects (e.g., impact of fetal thymic atrophy)	Holladay and Smith 1995
Ethanol	Latency, different developmental window effects	Giberson and Weinberg 1995, 1997; Giberson et al. 1997
Genistein	Different spectrum of effects	Guo et al. 2002
Lead	Dose sensitivity Differences in spectrum of effects Latency	Bunn et al. 2001a, 2001b, 2001c, Chen et al. 2004; Faith et al. 1979; Heo et al. 1996; Lee et al. 2001, 2002; McCabe et al. 1999; Miller et al. 1998; Snyder et al. 2000
Methoxychlor	Spectrum/severity of effects	White et al. 2005
Mercury	Dose sensitivity	Havarinasab et al. 2004; Hultman and Hansson-Georgiadis 1999; Silva et al. 2005
Nonylphenol	Spectrum/severity of effects	Karrow et al. 2004
Paracetamol	Dose sensitivity	Shaheen et al. 2005
T-2 toxin	Severity of effects (e.g., impact of fetal thymic atrophy)	Cooray and Jonsson 1990; Holladay et al. 1993b; Holladay et al. 1995
TCDD	Dose sensitivity	Gehrs and Smialowicz 1997, 1999; Gehrs et al. 1997; Smialowicz et al. 1994; Walker et al. 2004
Tributyltins	Dose sensitivity Spectrum/severity of effects	Tryphonas et al. 2004; Vos et al. 1990; Smialowicz et al. 1989
